# Phosphorylation Regulating the Ratio of Intracellular CRY1 Protein Determines the Circadian Period

**DOI:** 10.3389/fneur.2016.00159

**Published:** 2016-09-23

**Authors:** Na Liu, Eric Erquan Zhang

**Affiliations:** ^1^College of Life Sciences, Beijing Normal University, Beijing, China; ^2^National Institute of Biological Sciences, Beijing, China

**Keywords:** period length, phosphorylation, cryptochrome, subcellular localization, protein stability

## Abstract

The core circadian oscillator in mammals is composed of transcription/translation feedback loop, in which cryptochrome (CRY) proteins play critical roles as repressors of their own gene expression. Although post-translational modifications, such as phosphorylation of CRY1, are crucial for circadian rhythm, little is known about how phosphorylated CRY1 contributes to the molecular clockwork. To address this, we created a series of CRY1 mutants with single amino acid substitutions at potential phosphorylation sites and performed a cell-based, phenotype-rescuing screen to identify mutants with aberrant rhythmicity in CRY-deficient cells. We report 10 mutants with an abnormal circadian period length, including long period (S280D and S588D), short period (S158D, S247D, T249D, Y266D, Y273D, and Y432D), and arrhythmicity (S71D and S404D). When expressing mutated CRY1 in HEK293 cells, we show that most of the mutants (S71D, S247D, T249D, Y266D, Y273D, and Y432D) exhibited reduction in repression activity compared with wild-type (WT) CRY1, whereas other mutants had no obvious change. Correspondingly, these mutants also showed differences in protein stability and cellular localization. We show that most of mutants are more stable than WT, except S158D, T249D, and S280D. Although the characteristics of the 10 mutants are various, they all impair the ratio balance of intracellular CRY1 protein. Thus, we conclude that the mutations caused distinct phenotypes most likely through the ratio of functional CRY1 protein in cells.

## Introduction

To adapt to dramatic changes in environmental conditions, living organisms from fungi to humans have evolved an internal biological clock (1). In mammals, the circadian clock is an endogenously driven 24-h cycle affecting behavior, physiology, and metabolism (2). The core circadian oscillator is a transcription/translation feedback loop (TTFL) in which CLOCK and BMAL1 are activators that dimerize and promote the expression of *cryptochrome* (*CRY*) and *period* (*PER*) genes. After translation, CRY and PER form heterodimers and translocate to the nucleus where they act as repressors and inhibit their own expression. When nuclear localized CRY and PER proteins are degraded, the inhibition is relieved; the next circadian cycle is subsequently initiated (3).

Although the mechanism by which CRY and PER repress the transcriptional activation of CLOCK:BMAL1 is not well understood, post-translational modifications, such as the phosphorylation of CRY and PER proteins, are closely linked to the inhibition of transcription (4). The phosphorylation of PER proteins regulates their stability and their subcellular localization. In addition, different phosphorylation events can lead to phenotypes of opposite periodicity, such as the mutation in patients suffering from familial advanced sleep phase syndrome (FASPS) (5). In mammals, the CRY proteins, CRY1 and CRY2, are essential for the maintenance of circadian rhythms, and their absence results in arrhythmicity in constant darkness. In addition, CRY1 and CRY2 play different roles in regulating the circadian clock because mice lacking CRY1 or CRY2 exhibit short or long periods, respectively (6, 7). CRY1 and CRY2 are highly conserved proteins consisting of an N-terminal photolyase homology (PHR) domain, which binds to the flavin adenine dinucleotide (FAD) cofactor and divergent C-terminal tails (8). In CRY2, the phosphorylation of ser265 and ser553/ser557 may affect FAD positioning and electron transport, and proteasome degradation resulting in a shortened circadian period (9, 10). CRY1 phosphorylation in the PHR domain and C-terminal tail indicates that protein stability is linked to abnormal circadian rhythms (11, 12). Recent studies have shown that the stability of CRY proteins is regulated by two competing SCF E3 ligase complexes. The FBXL3 complex mediates degradation of CRY protein in the nucleus, while the FBXL21 complex protects CRY from FBXL3 degradation in the nucleus and promotes CRY degradation in the cytoplasm (13, 14). Although post-translational modifications of CRY1 are crucial for circadian rhythms, little is known about which CRY1 phosphorylation sites have the most impact. Therefore, we conducted a cell-based screen to identify phosphorylation residues in mCRY1 that rescue rhythmicity in *CRY1/CRY2* double-deficient cells (DKO cells) to better understand the role of phosphorylated CRY1 in clock function. We identified phosphorylation sites that cause long periods, short periods, or even arrhythmicity.

## Materials and Methods

### DNA Plasmids and Cells

P(*Cry1*)-CRY1 was constructed by replacing the P(*CMV*) promoter of pcDNA3.1-Cry1-Flag with the mCRY1 native promoter (1.5 kb) and the first intron (15). All of the mutations were generated using the KOD-plus-mutagenesis kit and confirmed by sequencing. HEK293 cells were purchased from the American Type Culture Collection (ATCC).

### Kinetic Bioluminescence Recording

Real-time circadian reporter assays were performed as previously described (16, 17). One the day prior transfection, approximately 3–5 × 10^4^ DKO cells were plated onto 35-mm culture dishes. Cells were cotransfected using the X-treme GENE HP DNA transfection reagent (Roche) with 1 μg of pGL3-P(*Per2*)-dLuc reporter plasmid and 50 ng of a CRY expression plasmid. Three days after transfection, the cells were treated with 0.1-mM dexamethasone (Sigma) for 2 h and then placed in XM medium as previously described (18). The kinetic bioluminescence was recorded using a Lumicycle luminometer (Actimetrics, Inc.) at 36°C.

### Luciferase Repression Assay

HEK293 cells were grown and transfected in 96-well plates. For transfection, 10 ng of the reporter plasmid pGL3-P(*Per2*)-dLuc was combined with 5 ng of a CRY expression plasmid, 10 ng of BMAL1, and 15 ng of the CLOCK plasmid. Empty vector pcDNA3.1 was added as necessary to obtain total DNA concentration of 200 ng per well. Twenty-four hours after transfection, cells were prepared for the Dual-Luciferase Reporter Assay System (Promega).

### Luciferase Complementation Assay

Luciferase complementation assay is used to determine the interaction of proteins (19). The N-terminal luciferase fragment was fused to the N-terminus of mCRY1 [wild type (WT) or mutant] and the C-terminal luciferase fragment to the C-terminus of mFBXL3 (or mPER2). mCRY1 (WT or mutant) and mFBXL3 (or mPER2) were co-expressed as fusion proteins with luciferase fragments in HEK293 cells. Twenty-four hours after transfection, cells were prepared for the Dual-Luciferase Reporter Assay System (Promega).

### Global Protein Stability Assay

Assays using the global protein stability (GPS) system were performed as described, with minor modifications (20). The GPS system was used to detect the stabilization of WT CRY1 and mutants. The lentiviral reporter construct contains a single promoter and an internal ribosome entry site (IRES) that permits the translation of two fluorescent proteins (DsRed and EGFP) from one mRNA transcript. DsRed served as an internal control, whereas EGFP was expressed as a fusion with our protein of interest. When integrated into the genome, the ratio of EGFP/DsRed can be quantified by fluorescence-activated cell sorting (FACS), producing a ratio that represents the stability of target proteins. The d1EGFP and d4EGFP represent half-life at 1 and 4 h, respectively.

### Subcellular Localization Assay

HEK293 cells were transfected with a plasmid encoding GFP-mCRY1 (WT or mutant). Twenty-four hours after transfection, the cells were stained with Hoechst 33258 (Sigma) and fixed with 4% paraformaldehyde in PBS. Samples were observed using Zeiss confocal LSM800 with a 63× water-immersion objective, and the data were analyzed using Image J software.

### Statistical Analysis

In all experiments, unless noted, error bars represent SEM (*n* ≥ 3 for each experiment). Statistical significance was determined using one-way ANOVA with Dunnett’s multiple comparisons test when comparing each mean to a control mean. All statistical analyses were performed using GraphPad Prism 6 (GraphPad Software, Inc., La Jolla, USA). *p < 0.05, **p < 0.01, ***p < 0.001, and ****p < 0.0001.

## Results

### The Phosphorylation of CRY1 Regulates Circadian Rhythms

**Figure 1:**
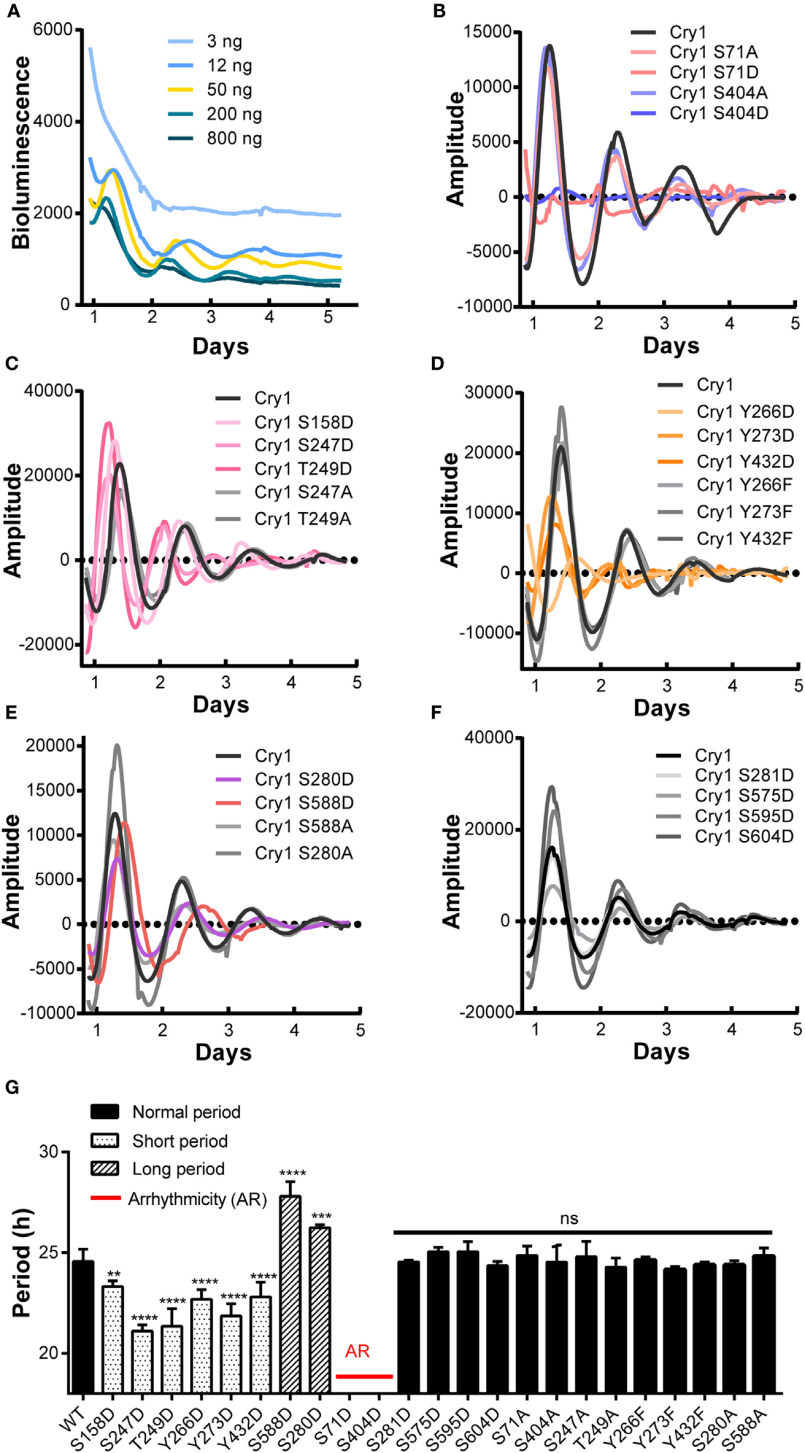
**A cell-based screen to identify critical phosphorylation residues on mCRY1 through rescuing rhythmicity in *Cry1^−/−^:Cry2^−/−^* fibroblasts (DKO cells)**. **(A)** Dosage-dependent rescue of circadian rhythms in DKO cells by mCRY1. The mCRY1 expression vector was cotransfected into cells with the P(*Per2*)-dLuc reporter vector. Three days after transfection, the cells were synchronized by dexamethasone treatment and then moved to luciferin-containing medium for 5–6 days of bioluminescence recording. **(B–F)** The P(*Per2*)-dLuc reporter rhythms (baseline subtracted) from DKO cells transfected with WT or mutant mCRY1, as noted in the legend. Experiments were performed as in **(A)**. **(G)** Quantitation of the period length from WT and mutant mCRY1 transfected cells that showed a distinct period phenotype as noted in the legend. Error bars represent SEM (*n* ≥ 5, **p < 0.01; ***p < 0.001; ****p < 0.0001, ANOVA).

The importance of core clock protein phosphorylation in the mammalian circadian system is widely accepted (4). However, little is known about the amount or location of phosphorylated CRY1 protein residues or how phosphorylation affects the molecular clockwork. In this study, we performed a cell-based screen to identify the phosphorylated residues in mCRY1 critical for rescuing rhythmicity in DKO cells. CRY1-mediated rescue of clock oscillation in DKO cells has been observed after transfection with CRY1 DNA concentrations ranging from 3 to 800 ng (Figure 1A). Our data show that 3 ng of CRY1 DNA was insufficient to rescue circadian rhythmicity, while 800 ng of CRY1 DNA restored circadian rhythms with low amplitude that were quickly dampened. Using 50 ng of CRY1 DNA, we rescued a circadian rhythmicity of approximately 24.6 h (Figure 1G).

**Figure 2:**
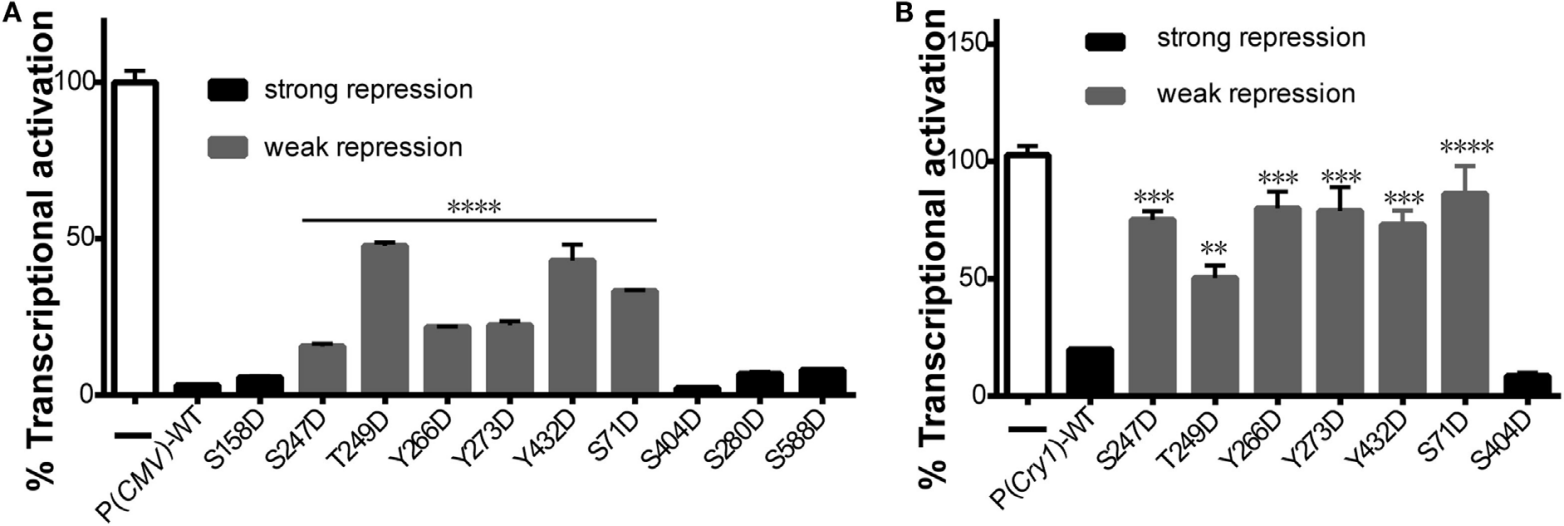
**Effects of mutant mCRY1 on BMAL1: CLOCK-induced transcriptional activation**. **(A)** HEK293 cells were cotransfected with BMAL1 (10 ng plasmid), CLOCK (15 ng) expression plasmid, and Per2-dluc (10 ng) reporter with WT or mutant mCRY1 (5 ng) as noted. Twenty-four hours after transfection, the cells were changed to luciferin-containing medium for end-point bioluminescence recording. The raw data were normalized such that the reporter control without mCRY1 (WT or mutant) transfection was equal to 100%. Compared with WT mCRY1, the mutants exhibited two profiles: strong repression with similar repression activity to WT and weak repression with a significant reduction in repression activity. Mean and error bars (SEM) of three independent transfections are shown (****p < 0.0001, ANOVA). Two additional experiments gave similar results. **(B)** DKO cells were cotransfected with Per2-dluc (10 ng) reporter and WT or mutant mCRY1 (50 ng) as noted. Experiments and data analysis were done as in **(A)**. Compared with WT, the mutants exhibited two profiles: strong repression with similar repression activity as WT and weak repression with a significant difference. Mean and error bars (SEM) of three independent transfections are shown (**p < 0.01, ***p < 0.001, ****p < 0.0001, ANOVA). Two additional experiments gave similar results.

**Figure 3:**
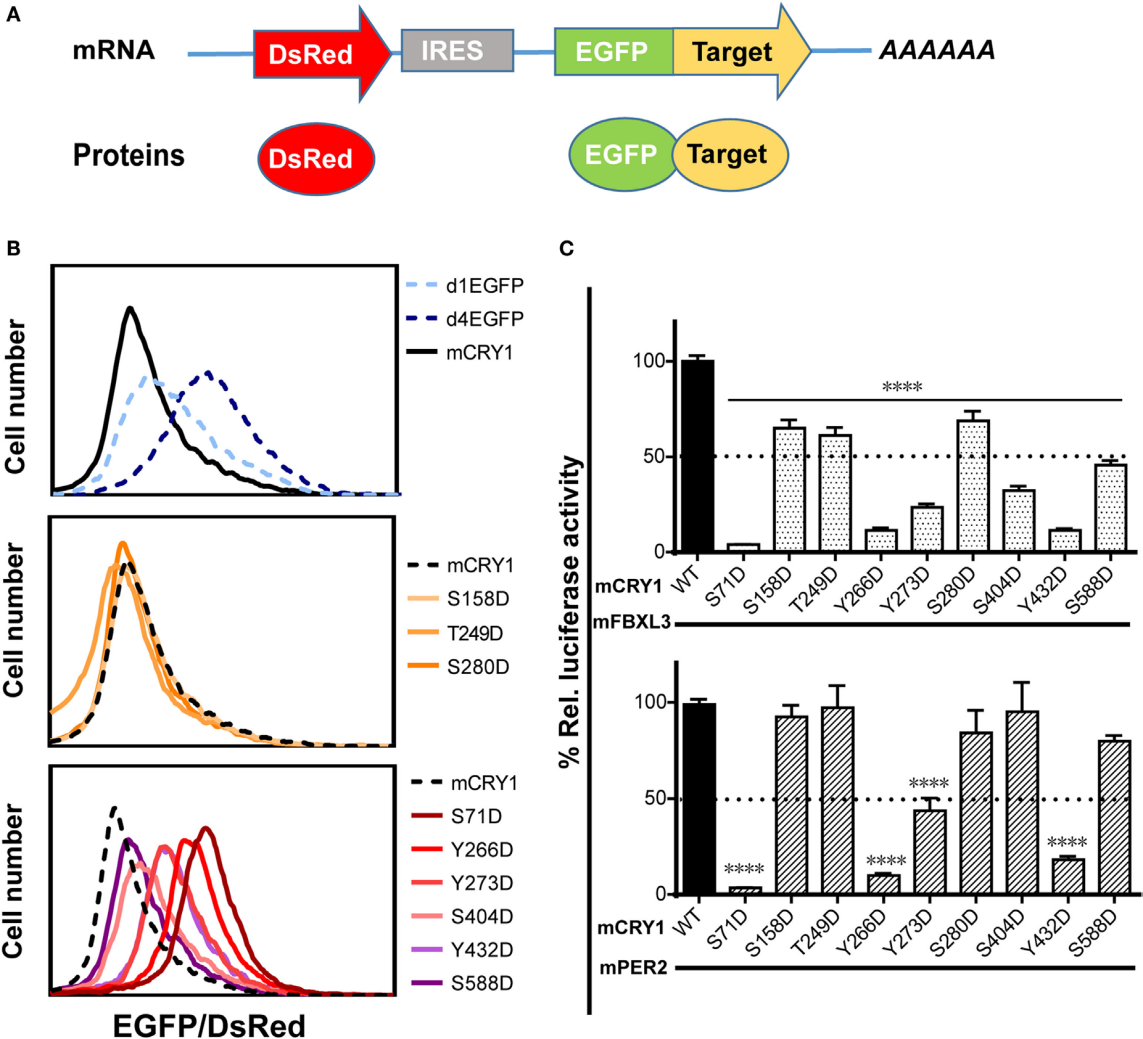
**Effects of phosphomimetic mutation on mCRY1 protein stability and interactions with FBXL3 and PER2**. **(A)** Global protein stability (GPS) reporter system. The DsRed–IRES–EGFP target element was cloned into a lentiviral vector, and the fluorescent reporter proteins were co-expressed from a single mRNA *via* an internal ribosomal entry site (IRES). **(B)** HEK293 cells were infected with lentivirus of pLv–DsRed–IRES–EGFP–mCRY1 (WT or mutant) for 24 h, and then the fluorescent protein signals were analyzed by flow cytometry. The EGFP/DsRed ratio acts as a reporter for stability of the expressed WT or mutant mCRY1. The d1EGFP and d4EGFP are markers for 1- and 4-h half-lives, respectively. **(C)** Luciferase complementation assay. mCRY1 (WT or mutant) and FBXL3 (or PER2) were co-expressed as fusion proteins with luciferase fragments in HEK293 cells. Experiments were done as in Figure 2A and the data presented relative to mCRY1 (WT)-mFBXL3 (or mPER2). Mean and error bars (SEM) of three independent transfections are shown (****p < 0.0001, ANOVA). Two additional experiments gave similar results.

**Figure 4:**
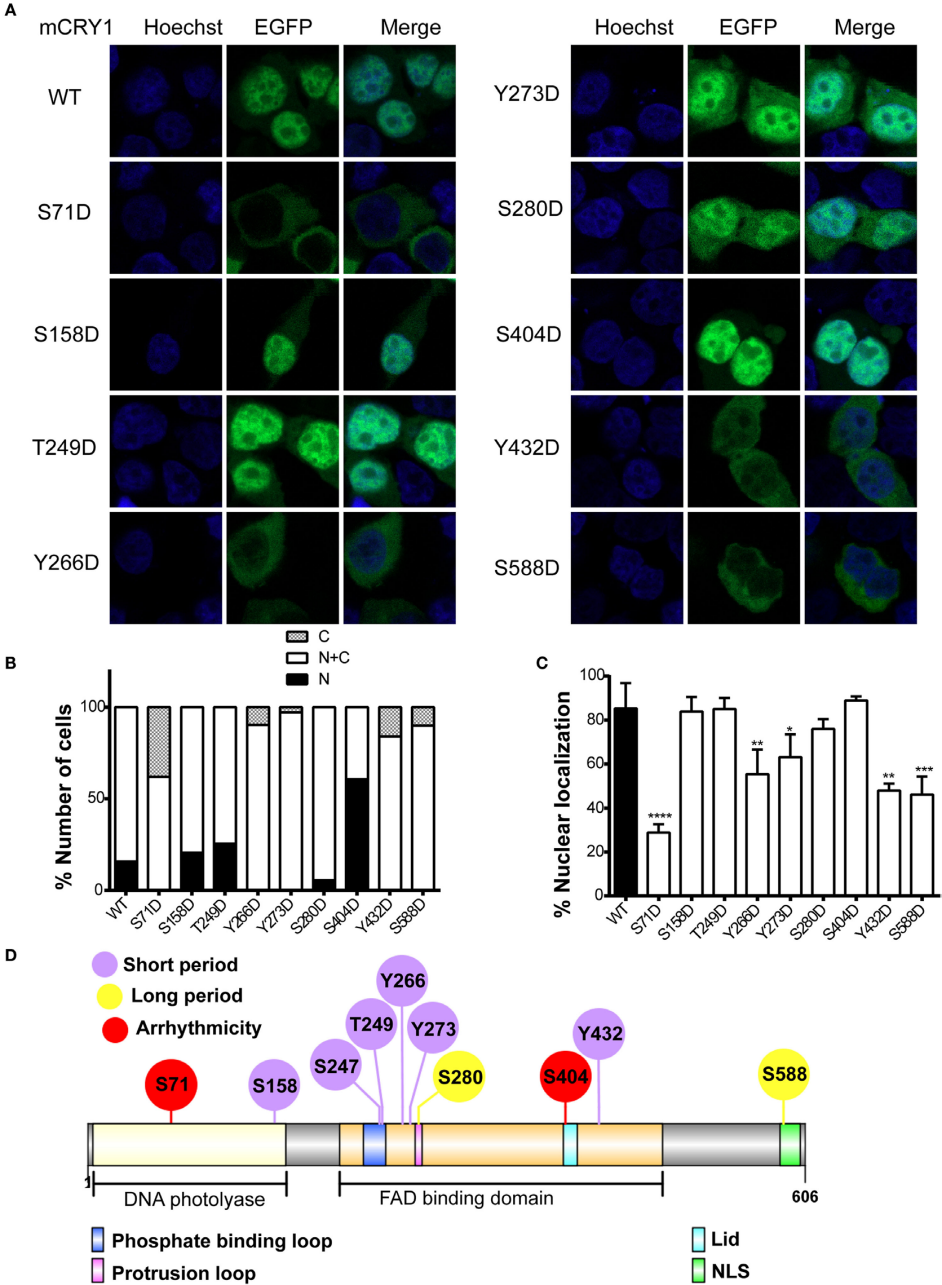
**Effects of phosphomimetic mutation on subcellular localization of mCRY1 protein**. GFP-tagged mCRY1 (WT or mutant) proteins were transiently overexpressed in HEK293 cells, and the subcellular distribution pattern of mCRY1 protein was analyzed. **(A)** Representative images of GFP-mCRY1 (WT or mutant) were detected by GFP fluorescence (green), and the nuclei were stained with Hoechst (blue). **(B)** Percentage of cells showing nuclear (N), nuclear–cytoplasmic (N + C), and cytoplasmic (C) staining as indicated in the plots. The ratio of cells with subcellular localization to the total transfected cells was analyzed by counting 100 cells three times in each experiment. **(C)** Percentage of colocalization of GFP-mCRY1 (WT or mutant) with nuclei. The 50 GFP-mCRY1 (WT or mutant)-expressing cells were analyzed by Image J software (Version 1.37c, NIH, USA). Mean and error bars (SEM) are shown (*n* = 3 for each experiment). Two additional experiments gave similar results (*p < 0.05; **p < 0.01; ***p < 0.001; ****p < 0.0001, ANOVA). **(D)** Location of the important phosphorylated sites on the liner protein functional regions of mCRY1. The FAD binding domain, which contains phosphate binding loop, protrusion loop, C-terminal lid (Lid), and nuclear localization signal (NLS), has been indicated in this schematic diagram. The diagram was constructed using Illustrator for Biological Sequences (IBS) software (24).

In our screen, phosphomimetics were created to identify potential phosphorylation sites in human, mouse, and rat (Table 1). We found 10 mutants with abnormal periodical phenotypes (Figures 1B–F). Our data demonstrate the following: (1) substitution of serine (S) 71 and 404 with aspartic (D) prevented the rescue of circadian rhythmicity (Figure 1B); (2) the phosphomimetic mutants for S158D, S247D, and T249D restored circadian rhythms with short periods (S158D, −1.3 h; S247D, −3.5 h; S249D, −3.3 h) (Figure 1G); (3) the mutants with Y266D, Y273D, and Y432D rescued circadian rhythms with short periods (Y266D, −1.9 h; Y273D, −2.7 h; Y432D, −1.8 h), low amplitudes, and quick damping (Figure 1G); (4) the S280D and S588D mutants restored circadian rhythms with long periods, especially S588D (S588D, +3.2 h; S280D, +1.6 h) (Figure 1G), which is consistent with previous reports (12); and (5) the phosphorylation of serines 281, 575, 595, and 604 had no obvious effect on circadian rhythms (Figure 1G). Thus, we established that most of the phosphorylation sites on CRY1 play distinct roles in the mechanism of the molecular clockwork.

**Table 1:** **Phosphorylated residues of CRY1 (mouse, human, and rat) are written in red**.

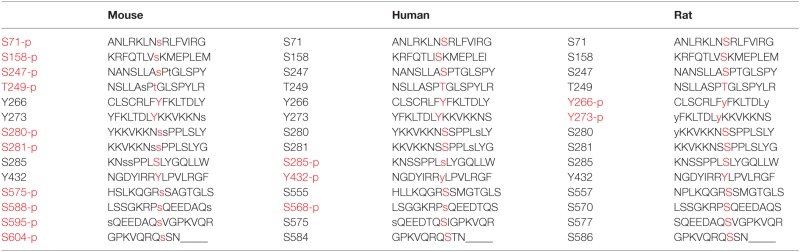

*The resource is from PhosphoSitePlus database (21)*.

To determine whether non-phosphorylation of the phosphorylated sites alters CRY1’s function, we generated non-phosphomimetic mutants of CRY1 with Ser/Thr/Tyr changed to Ala or Phe. Our results showed that all of the non-phosphomimetic mutants (S71A, S247A, T249A, S280A, Y266F, Y273F, Y432F, S404A, and S588A) exhibited lack of effect on the circadian period (Figure 1).

### Effects of Mutant mCRY1 on BMAL1: CLOCK-Induced Transcriptional Activation

To determine how CRY1 phosphorylation affects the molecular clockwork, we used a transcriptional assay to analyze the functional significance of the phosphomimetic mutants. Co-expression of CLOCK and BMAL1 stimulated E-box element-dependent transcription of a luciferase reporter gene in HEK293T cells, which was markedly suppressed by the expression of WT mCRY1 under the control of cytomegalovirus (CMV) promoter [P(*CMV*)] or the native mCRY1 promoter [P(*Cry1*)] (Figures 2A,B). We then constructed mutants by replacing phospho-acceptor Ser/Tyr/Trp residues with Asp, which mimics phosphorylation. The phosphomimetic mCRY1 mutants were used to determine the role of each residue in inhibiting transcription. Compared with WT mCRY1, the mCRY1 mutants fell into two phenotypic groups: strong repression (>60% of WT repression activity) that had similar repression activity to WT and weak repression (<20% of WT repression activity) with a significant reduction in repression activity (Figures 2A,B). The results showed that WT mCRY1 had strong repression activity that repressed the transcriptional activation to 3% driven by CMV promoter and to 20% driven by mCRY1 promoter. In addition, six Asp mutants (S71D, S247D, T249D, Y266D, Y273D, and Y432D) repressed the transcriptional activation to 16–50% and to 53–85%, respectively, under the control of CMV and mCRY1 promoter, exhibiting significant reductions in repression activity (*p* < 0.01, ANOVA). However, the other four Asp mutants (S158D, S280D, S404D, and S588D) exhibited no obvious change in repression activity (*p* > 0.05, ANOVA), although the repression activity of S404D was slightly stronger than WT (Figures 2A,B).

### Effects of Phosphomimetic Mutation on mCRY1 Protein Stability and Interactions with FBXL3 and PER2

Previous reports have shown that phosphorylation of mCRY1 at S247 does not affect protein stability (9). Therefore, we investigated whether phosphorylation of other residues that regulate mCRY1’s function (Figures 2A,B) alters protein stability. The GPS system utilizes an internally normalized fluorescent-based reporter system combined with FACS to detect real-time protein stability at the level of individual living cells (20, 22). GPS vectors expressed a single transcript encoding DsRed and EGFP target separated by an IRES (Figure 3A). The coding sequence for DsRed–IRES–EGFP–mCRY1 (WT or mutant) was cloned into a lentiviral vector. After infection by the lentivirus, HEK293 cells stably expressing DsRed and EGFP–mCRY1 (WT or mutant) were analyzed by flow cytometry. The EGFP/DsRed ratio acts as a reporter for stability of the expressed WT or mutant mCRY1. The d1EGFP (*t*_1/2_ = 1 h) and d4EGFP (*t*_1/2_ = 4 h) represent the half-life markers. Our results show that the half-life of WT mCRY1 is similar to that of d1EGFP (Figure 3B, top). The S158D, T249D, and S280D mutations exhibited no obvious change compared to WT mCRY1 (Figure 3B, middle). Surprisingly, the S71D, S404D, Y266D, Y273D, Y432D, and S588D mutations displayed half-lives longer than that of WT mCRY1 (Figure 3B, bottom), despite variations in the rescued period length (Figure 1G).

The crystal structure of mCRY1 reveals that binding sites for mPER2 and FBXL3, which partially overlap, are involved in transcriptional repression and protein stability (19, 23). To determine whether mCRY1 phosphorylation affects interactions with mFBXL3 and PER2, we used a luciferase complementation assay to determine how mFBXL3 or PER2 interact with phosphomimetic mCRY1 mutants (Figure 3C). WT or mCRY1 mutants and mFBXL3 (or PER2) were co-expressed as fusion proteins with N- and C-terminal luciferase fragments in HEK293 cells (19). Formation of mCRY1-FBXL3 (or PER2) complexes produces functional luciferase and that can be recorded in luciferin-containing medium. Data showed that, to varying degrees, all of the mutations reduced mFBXL3 binding. In particular, the S71D, Y266D, Y273D, S404D, and Y432D mutations drastically reduced mFBXL3 binding to 4, 11, 23, 32, and 11%, respectively (Figure 3C, top). By contrast, mPER2 binding was unaffected by the S158D, T249D, S280D, S404D, and S588D mutations, while the S71D, Y266D, Y273D, and Y432D mutations weakened the interactions with mPER2 to 4, 10, 43, and 18%, respectively (Figure 3C, bottom). We conclude that phosphomimetic mutations affect the stability and transcriptional repression activity of mCRY1 by antagonizing with mFBXL3 and PER2.

### Effects of Phosphomimetic Mutation on mCRY1 Protein Subcellular Localization

The stability of CRY1 protein is regulated by two competing SCF E3 ligase complexes: FBXL3 mediates degradation in the nucleus, while FBXL21 protects CRY1 in the nucleus and facilitates degradation in the cytoplasm (13, 14). Therefore, we sought to determine whether phosphomimetic mutations alter the subcellular localization of mCRY1. To determine the subcellular distribution pattern of the mutants, we generated a GFP-tagged mCRY1 (WT or mutant) expression construct. Representative images of GFP-mCRY1 (WT or mutant), as detected by GFP fluorescence, are shown in Figure 4A. The ratio of cells with subcellular distribution and the colocalization of GFP-mCRY1 (WT or mutant) proteins with nuclei are shown in Figures 4B,C. In transient transfection assays using HEK293 cells, the mutants were predominantly localized in the nucleus and cytoplasm. However, 5–65% of S158D, S249D, S280D, or S404D-GFP were localized exclusively in the nucleus, similarly to WT, with a colocalization efficiency of more than 75%. In contrast, 6–42% of S71D, Y266D, Y273D, Y432D, or S588D-GFP were only observed in the cytoplasm, with a nuclear colocalization efficiency of less than 63%, especially S71D (~29%) (Figures 4B,C). Based on these data, we conclude that the phosphorylation of mCRY1 at amino acid sites S71, Y266, Y273, Y432, and S588 alter the subcellular localization that is critical for the rhythmicity of circadian clock.

Although the mutants S158D, S247D, and T249D showed similar periodical phenotypes with Y266D, Y273D, and Y432D, the subcellular location of the proteins had no obvious change, suggesting that the mechanisms were different. Indeed, the identified phosphorylated sites were located in different functional regions of mCRY1 (Figure 4D), supporting our conclusion that the phosphorylation of these sites with different repression activity, protein stability, and subcellular location results in various periodical phenotypes by distinct mechanisms.

## Discussion

In the basic TTFL model, both positive and negative regulatory elements are important for generating the autoregulatory feedback loop. Post-translational regulation of the activity, degradation, and localization of these regulators, most notably phosphorylation influences the circadian rhythms (4, 25). Although many CRY1phosphorylation sites have been identified, their contribution to clock function was unclear. We conducted a cell-based screen in CRY-deficient (DKO) cells and identified 10 phosphomimetic mutants of mCRY1 that induce abnormal circadian periods, including long period (S280D and S588D), short period (S158D, S247D, T249D, Y266D, Y273D, and Y432D), and even arrhythmicity (S71D and S404D). The period length of the circadian clock in cells is complicated because many genes participate in regulating the circadian period (11, 26–28). In this study, we identified mutations that alter repression activity, protein stability, and cellular localization, suggesting that distinct mechanisms regulate each phenotype.

Previously, we have demonstrated that the proper ratio of intercellular CRY proteins determines the normal clock period length (17). In this study, we further determined that the ratio of functional CRY1 protein is regulated by phosphorylation in cells and the ratio imbalance disrupts circadian rhythmicity, although the mechanisms are different (Table 2). The S280D mutation displayed a long period, similar to that of S588D [previously reported in Ref. (12)]. We found that phosphorylation at S588 decreased nuclear protein localization and weakened interactions with FBXL3, increasing the protein stability. This may be due to the position of the S588 residue, which is near the NLS sequence of the C-terminal tail of mCRY1. The crystal structure of mCRY1 (19) shows that FBXL3, but not PER2, binds across the protrusion loop (S280) and the phosphate-binding loop (S247 and T249) (Figure 4D). Consistently, the S280D and T249D mutations reduced FBXL3 binding but did not affect PER2 binding (Figure 3C). In addition, the phosphate-binding and protrusion loops, with conformational flexibility, constrict the approach to FAD, which is critical to CRY1’s functions. Phospho-Ser-mimicking mutations at this region may enforce the phosphate-binding conformation to tune FAD, ultimately leading to a change in mCRY1 protein function (8). Introduction of negative charge (Asp) to site S158, located in the surface region (19), may restructure and/or disorder the structural conformation between the phosphorylation site and nearby amino acid residues, affecting mCRY1’s clock function. The Y266D, Y273D, and Y432D mutations displayed similar phenotype (short period, Figure 1D), weaker interactions with FBXL3 and PER2, and lower nuclear colocalization efficiency compared with WT-mCRY1. Interestingly, although the S71D mutant and S404D mutant are arrhythmic, their molecular character is very different. The S71D mutant displayed almost no interactions with FBXL3 and PER2 and high colocalization efficiency with the cytoplasm. Regardless of a subtle difference with previous reports (11), in which the S71D mutation increased interaction with FBXL3, phosphorylation at S71 is crucial for regulating circadian period. In addition, phosphorylation at these sites weakens the binding with PER2, slowing the rate of nuclear translocation and decreasing the concentration of functional protein (3, 29). Nuclear transport of the PER/CRY complex is reported to be one of the most important mechanisms for period regulation, as shown in the recent report on nuclear importin KPNB1 (30). In contrast, the phosphomimetic mutation of S404, located in the C-terminal lid, did not rescue DKO cells with hyper-repression activity (Figures 2A,B) and hypo-interactions with FBXL3 increasing protein stability. Neither did it affect binding to PER2 nor nuclear colocalization. We hypothesized that the S404D mutant binding to FBXL3 was very weak, thus slowing degradation, prolonging interactions with the BMAL1/CLOCK complex, and ultimately preventing the relief of inhibition and initiation of the next circadian cycle (31).

**Table 2:** **Phosphorylation regulating the ratio of intracellular mCRY1 determines the circadian period length by different mechanisms**.

Phosphomimetic mutants	Period phenotype	Repression activity	Protein stability	Nuclear colocalization	Potential mechanism
S71D	AR	↓	↑	↓	The mutant cannot enter the nucleus, as the binding to PER2 is too weak
S404D		↑	↑	N	The degradation is weak, as the binding to FBXL3 is weak

S158D	S	N	N	N	Introduction of negative charge to the surface region alters the interaction with other proteins
S247D		↓	—	—	The mutants enforce the phosphate-binding conformation to tune FAD, ultimately leading to change in mCRY1 protein function
T249D		↓	N	N	
Y266D		↓	↑	↓	Lower nuclear localization efficiency may be due to weak interaction with FBXL3 and PER2
Y273D		↓	↑	↓	
Y432D		↓	↑	↓	

S280D	L	N	N	N	The binding to FBXL3 is weak, as S280 is located in protrusion loop, which interacts with FBXL3 and constricts access to FAD
S588D		N	↑	↓	The ratio of nuclear protein is decreased. This may be due to S588 nearing the NLS sequence

*AR, arrhythmicity; S, short period; L, long period; ↑, increase; ↓, decrease; N, no obvious change; —, no result*.

In summary, we identified critical CRY1 sites where mutations disrupted circadian rhythmicity. Although some enzymes correspond to specific modifications in mCRY1, such as MAPK at S247 and AMPK at S71 and S280 (9, 11), the enzymes that modify the other sites are unknown. In addition, the effect of modifiers on rhythmicity varies after blocking (28), but how the enzymes work on the circadian clock components remains unclear. Our data indicate that key modifiers of CRY1 directly regulate the ratio of functional CRY1 protein by distinct mechanisms that determine the circadian rhythmicity, providing new insights on regulation of the circadian period.

## Author Contributions

NL and EZ conceived the study, designed the experiments, and analyzed the data. NL performed the experiments. NL and EZ wrote the manuscript.

## Conflict of Interest Statement

The authors declare that the research was conducted in the absence of any commercial or financial relationships that could be construed as a potential conflict of interest.
